# A Phase II study of Viagenpumatucel-l (HS-110) in combination with low-dose Cyclophosphamide versus physician's choice in patients with advanced non-small cell lung cancer

**DOI:** 10.1186/2051-1426-2-S3-P82

**Published:** 2014-11-06

**Authors:** Roger B Cohen, John Nemunaitis, Nashat Gabrail, Lyudmila Bazhenova, Taylor H Schreiber, Melissa Price

**Affiliations:** 1 University of Pennsylvania School of Medicine, Philadelphia, PA, USA; 2 Mary Crowley Medical Research Center, Dallas, TX, USA; 3 Gabrail Cancer Center, Canton, OH, USA; 4 University of California San Diego, CA, USA; 5 Heat Biologics, Durham, NC, USA

## Background

Viagenpumatucel-L (HS-110) is an allogeneic cell-based therapeutic cancer vaccine for the treatment of advanced NSCLC. The vaccine is composed of a cell line expressing a defined repertoire of tumor antigens (MAGE-A3, NY-ESO-1, LAGE-1, and others) that are chaperoned by a modified, secretable, heat shock protein (gp96-Ig) with dual antigen binding and adjuvant activity. Importantly, gp96-Ig delivers these cell-derived antigens directly to a patient's own antigen presenting cells and shuttles those antigens to MHC-I via the cross-presentation pathway. This preferential trafficking to MHC-I leads to exclusive activation of CD8+ cytotoxic T cells. In addition, the specific delivery of cell-derived antigens by gp96-Ig enables vaccination across MHC barriers and leads to potent CD8+ T cell activation in response to physiologic antigen concentrations (femto-molar). These elements are believed to provide several advantages over previous peptide vaccines by providing an opportunity for protection against a broad spectrum of tumor antigens and therefore addressing the underlying genetic/antigenic heterogeneity of NSCLC within individual tumors. In addition, delivery of antigens to MHC-I and activation of CD8+ cells is proposed to provide a unique advantage compared to other cell-based vaccines, wherein antigens are preferentially processed through the exogenous pathway and displayed on MHC-II.

## Methods

This is a Phase II study of 123 patients who have failed two prior lines of therapy for advanced NSCLC. Patients randomized to the experimental group will be treated during an induction phase with combination Viagenpumatucel-L weekly for 12 weeks (10^7 ^cells/dose) and metronomic Cyclophosphamide (50 mg daily for 7 days; one week on, one week off, for 12 weeks), followed by monotherapy Viagenpumatucel-L in the maintenance phase on Day 1 of a three-week cycle. Patients randomized to the control group will receive one of the single-agent physician's choice regimens (e.g., Gemcitabine, Vinorelbine, Erlotinib, or Taxane) on a nominal three-week schedule with dose and route according to investigator's standard practice. Treatment will continue for up to one year or until progression by immune-related response criteria (irRC). The primary endpoint is overall survival. Secondary efficacy evaluations include determination of overall response, progression-free survival, and disease control rate, by both irRC and RECIST. Analyses of immunologic response in peripheral blood and antigen expression analysis in tissue are also planned. Clinical trial information: NCT02117024.

